# Generalisation within specialization: inter-individual diet variation in the only specialized salamander in the world

**DOI:** 10.1038/srep13260

**Published:** 2015-08-21

**Authors:** Andrea Costa, Sebastiano Salvidio, Mario Posillico, Giorgio Matteucci, Bruno De Cinti, Antonio Romano

**Affiliations:** 1 Consiglio Nazionale delle Ricerche, Istituto di Biologia Agroambientale e Forestale (CNR-IBAF), Area di Ricerca Roma 1, Via Salaria km 29, 300-00015. Monterotondo (RM) (Italy); 2 Dipartimento di Scienze della Terra, dell’Ambiente e della Vita, Università di Genova, Corso Europa 26, I-16132. Genova (Italy); 3 Corpo Forestale dello Stato, Ufficio Territoriale Biodiversità di Castel di Sangro, Via Sangro, 45-67031. Castel di Sangro (AQ) (Italy)

## Abstract

Specialization is typically inferred at population and species level but in the last decade many authors highlighted this trait at the individual level, finding that generalist populations can be composed by both generalist and specialist individual. Despite hundreds of reported cases of individual specialization there is a complete lack of information on inter-individual diet variation in specialist species. We studied the diet of the Italian endemic Spectacled Salamander *(Salamandrina perspicillata*), in a temperate forest ecosystem, to disclose the realised trophic niche, prey selection strategy in function of phenotypic variation and inter-individual diet variation. Our results showed that *Salamandrina* is highly specialized on Collembola and the more specialized individuals are the better performing ones. Analyses of inter-individual diet variation showed that a subset of animals exhibited a broader trophic niche, adopting different foraging strategies. Our findings reflects the optimal foraging theory both at population and individual level, since animals in better physiological conditions are able to exploit the most profitable prey, suggesting that the two coexisting strategies are not equivalent. At last this species, feeding on decomposers of litter detritus, could play a key role determining litter retention rate, nutrient cycle and carbon sequestration.

Specialization is a widespread topic in ecology and evolutionary biology. Even if definitions of what should be called specialization and its boundaries have been debated by researchers for a long time^1^,^2^,^3^, there is a certain tendency in defining as specialist a species that utilizes a narrow range of resources. Similarly to the different definitions of ecological specialization, the set of resources on which a species can specialize is equally varied: indeed ecological specialization is inferred, among others, on habitat, space, shelter and food. Furthermore ecological specialization can be studied at different scales in space and time. As a matter of fact ecological specialization is reported at different biological levels^3^: population, species, community and, recently, specialization has been also studied at the individual level^4^. Nevertheless, despite discrepancies in the definitions of specialization and the coexistence of different and opposite views in these topics, many authors agree that specialist species are potentially more vulnerable to habitat fragmentation than generalists^5^,^6^,^7^ and that they are more temporally variable and more prone to extinction. Furthermore, for the aforementioned reasons, detecting patterns of specialization in itself may be a helpful tool for understanding and advising populations’ declines and to address proper conservation measures.

Specialization is almost always inferred at species or population level, and only recently a new approach to the study of specialization emerged: the study of inter-individual diet variation^4^. From this point of view the trophic niche of a population is seen as the result of the sum of the trophic niche of all individuals. As a matter of fact, the trophic niche of every single individual may be very different, at the point that a generalist population may be composed by individuals with different degrees of specialization, that feed on different prey types and adopt different trophic strategies. Intra-population niche variation is usually inferred as a result of different processes related to the individual characteristics^4^,^8^: i) sexual dimorphism and sexual differences in resource use, ii) ontogenetic diet shift, and iii) resource polymorphism. Evidences of individual specialization are mainly based on dietary data, but evidences for habitat selection and other niche axes are also documented, ranging from Plants to Mammals, through Crustaceans, Gastropods, Fishes, Amphibians and Birds^8^,^9^. Despite the fact that individual specialization has been observed, in many cases *ante-litteram*, in nearly 200 species regarding the use of some resource^9^, there is a complete lack of evidence for inter-individual resource variation in species or populations that are true specialists. The concept of “individual specialization” seems to exclude *per se* the possibility of studying inter-individual diet variation in populations where the emerging trophic strategy leads to specialization: furthermore an “individual specialist” is defined “as an individual whose niche is substantially narrower than its population’s niche for reasons not attributable to its sex, age or discrete morphological group”^8^.

In this study we analyze the diet variation of the Italian endemic Spectacled Salamander (*Salamandrina perspicillata* Savi, 1821) which, on the basis of previous studies^10^,^11^, seems to be a specialist predator on Collembola, at least in autumn. The aims of this study are threefold: first we investigate more in detail the diet of *Salamandrina perspicillata* to verify if the specialization observed in previous studies is a species-specific trait, as well as its seasonal trend. Second we evaluated the prey selection strategy, relating the realized trophic niche to trophic availability, and its relationships with phenotypic variation. Third, we evaluate, for the first time, the inter-individual level of diet variation in a specialist predator and its seasonal variation, turning the classic question “Are there, within a generalist population, any specialized individuals?” into the less common “Are there, within a specialist population, any generalist individuals that adopt a different foraging strategy?”.

## Results

### Sampling salamanders and their prey

During our surveys we captured and processed 187 Salamanders (120 and 67 in Autumn and Spring, respectively, for a total of 117 males, 70 females) from two neighboring populations and all of them provided stomach contents, from which we obtained 3912 invertebrates representing 17 taxa in Autumn and 18 in Spring. All the individuals processed in this study are reproductive adults; their body size lies within a narrow range [40.40 (3.07) – Mean body length (SD)], and juveniles were not included in the analyses. Environmental sampling produced a total number of 3824 invertebrates (1488 and 2336 in Autumn and Spring, respectively) representing 26 taxa in Autumn and 21 in Spring. Since our interest is to describe the trophic strategy at species-level we verified, through multivariate analysis of similarity (ANOSIM), i) that the invertebrate community composition was similar between sites during both seasons (Autumn; R = −0.04; p = 0.691 – Spring; R = −0.05; p = 0.801), ii) that the two populations had similar diet composition (Autumn; R = −0.003; p = 0.654 – Spring; R = −0.029; p = 0.830) and then we merged the data to analyze prey selection. The complete dataset both for stomach contents and for environmental sampling are shown in Table 1.

### Is *Salamandrina* a specialist predator?

**Figure 1:**
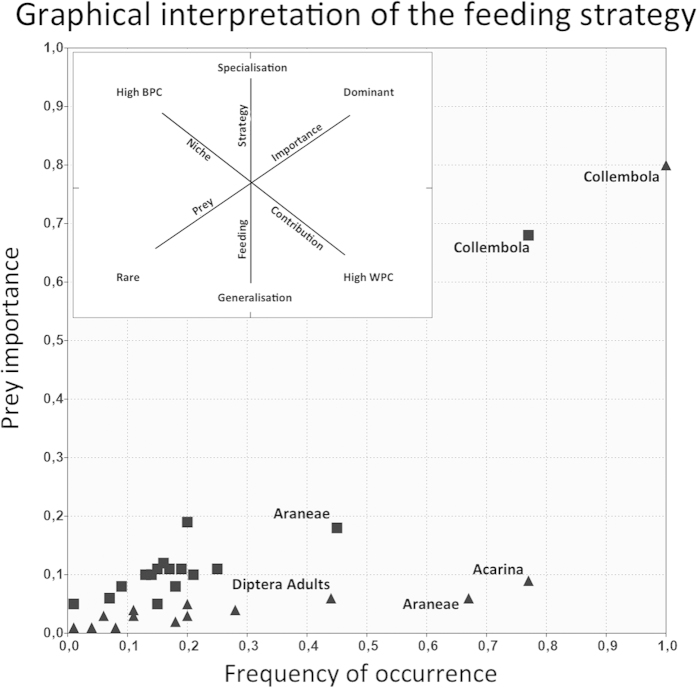
Costello’s modified graphical interpretation of the foraging strategy (Amundsen *et al.*, 1992). Autumn data are represented by squares, while spring data are represented by triangles. Labels of prey categories with both values of Prey importance and Frequency of occurrence lower than 0.30 are not shown.

With regard to the trophic niche width of *Salamandrina,* the diversity in the diet composition was significantly higher (t-test: p < 0.001) in Autumn (Shannon-Weaver index = 1.16 – Evenness = 0.40) than in Spring (S-W index = 0.83 – Evenness = 0.28), and the diversity, for the invertebrate community, significantly differed between seasons (t-test: p < 0.001) and was higher in Autumn (S-W index = 2.23 – Evenness = 0.68) than in Spring (S-W index = 1.86 – Evenness = 0.61). There was no significant evidence of different prey use between sexes (ANOSIM: Autumn; R = −0.06; p = 0.711 – Spring; R = −0.07; p = 0.842). The graphical representation of the feeding strategy (Fig. 1) confirmed that *Salamandrina*, in both seasons, behaves as a specialist predator on Collembola. Furthermore, the graphical analysis suggests that *Salamandrina* has a narrow trophic niche, and Collembola were the bulk of the realized trophic niche, along with Araneae and Acarina, while these contributed to a lesser extent. Even if *Salamandrina* appears to be specialized on Collembola in both seasons, the intensity of specialization is even higher in Spring than in Autumn: indeed all salamanders fed on Collembola in Spring.

### How does *Salamandrina* select prey?

**Figure 2:**
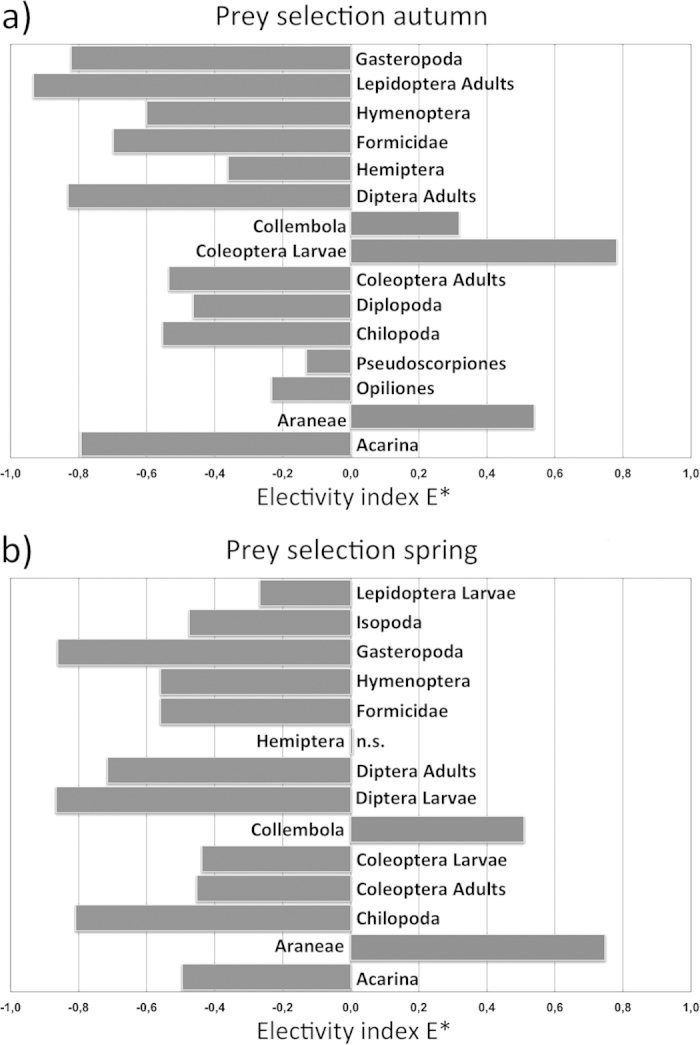
Vanderploeg and Scavia’s (1979) electivity index E* for the most representative prey categories in autumn (**a**) and spring (**b**). n.s. = not significant values.

*Salamandrina* positively selects only few prey categories, within a relatively large array of available taxa (Fig. 2). In both seasons, Collembola and Araneae are positively selected, while all other prey categories are clearly used less than expected based on their availability (e.g. Acarina). In Autumn, *Salamandrina* showed a positive selection for more prey categories (Collembola, Coleoptera larvae, Araneae) compared to Spring (Aranea, Collembola).

Resource selection probability functions (RSPF) were applied to the three main selected prey types: Collembola, Araneae and Acarina. Collembola accounted for the highest estimated probability of use [0.83 (0.21) – Mean probability of use (SD)], while the best model included season, snout-vent length (SVL), and body condition index (SMI). Probability of use was higher in Spring than in Autumn [13.04 (13.10; 14.64) – Beta’s estimates (95% Confidence Intervals)] and was positively influenced by SVL [0.46 (0.31; 0.62)] and SMI [0.52 (0.39; 0.72)]. The estimated probability of use for Araneae [0.01 (0.002) – Mean probability of use (SD)] and Acarina [0.07 (0.023) – Mean probability of use (SD)] was remarkably lower than that estimated for Collembola. Acarina probability of use resulted to be influenced by season [0.73 (0.40; 1.21)] but negatively affected by SVL [−0.16 (−0.9; 0.03)]. Similarly, Araneae’s probability of use was influenced by season [−0.80 (−1.11; −0.59)], but with an opposite trend in comparison to Collembola and Acarina. Furthermore the use of Araneae resulted to be negatively affected by SMI [−0.19 (−0.26; −0.10)] and index of foraging intensity, FORI [0.42 (−0.56; −0.28)].

### Is there an inter-individual diet variation?

**Figure 3:**
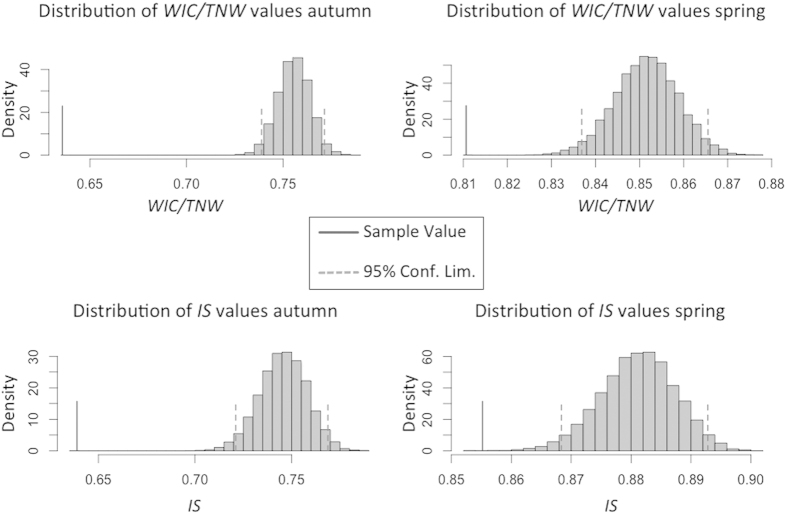
Histograms of distributions of within-individual component/trophic niche width (WIC/TNW) ratio and individual specialization (IS) indices obtained by Monte Carlo resampling procedure, both for autumn and spring data. Vertical broken lines show the 95% confidence limits of the simulated distribution, while the vertical solid line shows the actual index value for the original data.

The Total Niche Width (TNW) of the population in Autumn is wider than in Spring (TNW = 1.16—0.83; in Autumn and Spring, respectively). The Within Individual Component of the trophic niche (WIC) resulted similar between seasons (WIC = 0.74—0.67; in Autumn and Spring respectively), while the Between Individual Component (BIC) decreases from Autumn to Spring (BIC = 0.42—0.15; in Autumn and Spring, respectively). The value of the ratio WIC/TNW is lower in Autumn (0.63) than in Spring (0.81), and in both season it’s statistically significant (Monte Carlo resampling; p = 0.001) (Fig. 3). The population has been divided into two groups at the proportional similarity index (PSi) value 0.73 in autumn and 0.87 in spring; obtaining two sub-groups on the basis on the individual specialization (48 and 72 individuals in Autumn; 33 and 34 individuals in Spring). In Autumn individuals with low values of PSi had significantly lower values of SMI (Mann Withney; U = 1159, p < 0.001) while during Spring the two groups had similar values (Mann Withney; U = 438, P = 0.12). SVL did not differ significantly between groups in both seasons. The diet of groups characterized by low values of PSi had significantly higher diversity (Mann-Withney; U = 1255, p = 0.006 in Autumn; U = 260, p < 0.001 in Spring) and evenness (Mann-Withney; U = 1084, p < 0.001 in Autumn; U = 220, p < 0.001 in Spring). At the same time the “low PSi” groups accounted for a smaller FORI (Mann-Withney; U = 380, p < 0.001 in Autumn; U = 180, p < 0.001 in Spring) and showed a lower relative abundance of Collembola in their stomachs (Mann-Withney; U = 702, p < 0.001 in Autumn; U = 245, p = 0.002 in Spring) in both seasons. Complete data for the aforementioned analyses, including means and standard deviations of the two subgroups, are presented in Table 2.

## Discussion

Prior to this study, *Salamandrina* was known to behave as a specialist predator on Collembola, especially in Autumn^10^,^11^, while during the transition from Autumn to Spring a shift towards a more generalist foraging strategy was described^10^. However in the present study, based on a larger sample size, *Salamandrina* behaves as a specialist predator in both seasons. Furthermore in Spring the realized trophic niche is narrower and the degree of specialization is higher than in Autumn. This pattern of variation, related to seasonal changes in trophic availability is well known and fully reflects the Optimal Foraging Theory^12^. In fact Optimal Foraging Theory states that individuals are capable to categorize prey items on the basis of their net energetic value (net gain of energy considering the cost of capture, handling and digesting) and always select those prey that guarantee the highest energy intake per time unit. When more profitable prey categories become scarce, an expansion of the trophic niche is expected, and other prey categories are also exploited, maximizing the energy intake per time unit.

The slightly contrasting results we obtained from available data^10^ may only be apparent. In the other studied site^10^ *Salamandrina* shares the environment with another terrestrial salamander, the plethodontid *Speleomantes strinatii.* As reported by those authors^10^ the dietary pattern showed in their study population, contrasting the Optimal Foraging Theory, may be strongly influenced by the syntopy of two (or more) species that have similar ecological and trophic requirements and niche partitioning may play a key role in their long-term coexistence^13^,^14^,^15^. Conversely, *Salamandrina* is the only salamander occurring in our study area and our results revealed its trophic habits when trophic competition does not occur.

Results from the Electivity Index E* show how *Salamandrina* selects only few prey categories from a highly diverse pool of resources available in the environment. Collembola, Araneae and Coleoptera larvae are selected during the Autumn, while in Spring only Araneae and Collembola are selected. Furthermore, in agreement with Optimal Foraging Theory, the fact that some prey categories, such as Acarina, that are particularly abundant in the environment, are avoided in both seasons should suggests that those prey are categorized by *Salamandrina* as a low profitable resource. Indeed, even if the size and energy content of Collembola and Acarina is similar^16^, Acarina have harder bodies and are only partially digested by salamanders^11^, providing a lower energy intake per volume unit.

**Figure 4:**
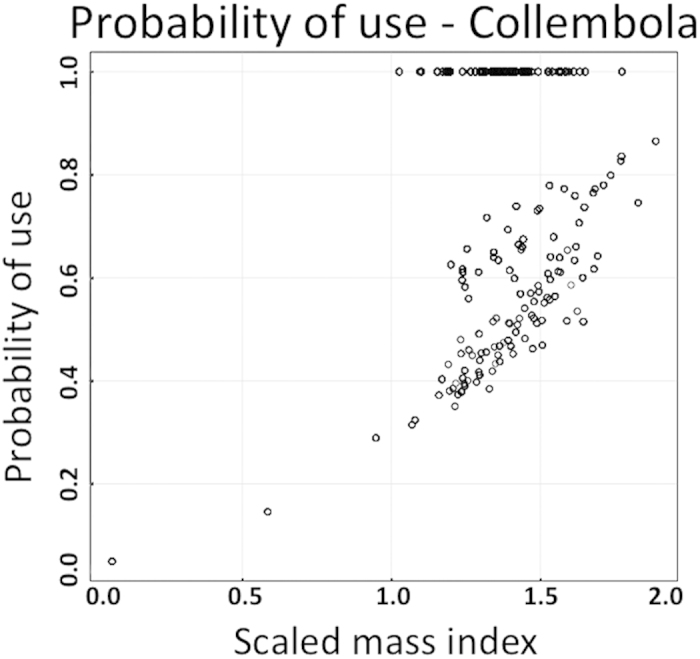
Scatterplot of the probability of use of Collembola, obtained from resource selection probability functions (RSPF), in relation to body condition index (SMI).

Collembola are little armored and therefore highly energetic^17^ but, at the same time, hard to catch because they evolved their furcula as an escape mechanism to avoid predators, being able to jump like “miniature kangaroos”^18^. Thus, our findings concerning the seasonality effect, which mainly deal with prey availability, and the phenotypic variation of salamanders, may be regarded as a balance between the energy content of a given food and the difficulty in obtaining it. Larger salamanders, and in a better physiological condition, have higher probabilities of consuming Collembola (Fig. 4), while smaller and under-performing individuals had high probabilities of selecting Acarina and Araneae.

Diet variation among individuals may arise from their differences in experience and foraging ability^8^,^19^,^20^. The observed inter-individual variation in prey selection could be explained by Optimal Foraging Theory, since phenotypic variation may play an important role in determining prey selection. Different individuals may have different abilities to detect or consume prey and those differences will lead to a different categorization of which one is the most profitable prey. Therefore, individuals with lower fitness, for genetic or environmental causes, are not able to exploit the most profitable prey category, for instance due to intraspecific competition when the preferred resource is scarce, and shift their selection on other prey categories.

Animal populations are commonly composed of both generalist and specialist individuals with the latter representing a small subset of the whole population^20^. We observed for the first time a different pattern, where generalists are a smaller subset within a population mainly composed by specialized individuals. Diet specialization in *Salamandrina* seems to be the rule, while inferior performing individuals shift their diet, in particular during the season with low trophic availability, toward a wider trophic niche that included low-energy but easy-to-catch prey. Indeed in other taxa, specialist individuals had higher foraging success^21^, a feature related to a higher fitness leading to a higher reproductive success^21^,^22^. Among amphibians diet specialization was known so far only for anurans^23^,^24^ and it is often linked with the evolution of aposematism^25^. Our study revealed that *Salamandrina perspicillata* is the only salamander in the world exhibiting a true diet specialization. Among Urodela diet specialization is known only for a single population of the Italian Crested Newt (*Triturus carnifex*, a generalist species^26^,^27^), living in a deep flooded karstic sinkhole, that in summer prey almost exclusively on the pre-imaginal stages of the small China-mark, *Cataclysta lemnata*^28^. Consequently our study and the previous one^10^, for populations separated by 520 km as the crow flies, reveal that diet specialization in *Salamandrina* is a species-level habit, highlighting the existence of the first urodelan in the world with a truly specialized diet. Specialization at individual level intrigued ecologist for decades but it has been studied only within generalist populations/species. We wanted to contribute to this debated topic by providing a different perspective, i.e. studying what emerged by analyzing the feeding habits of individuals in a specialized species. On the whole the population we studied consist by two subgroups of salamanders, the more performing one highly specialized on a very energetic but hard to catch prey type. The less performing animals instead shift their trophic strategy towards more easy-to-catch but less energetic taxa, in particular during the season with lower trophic availability. In our study case, specialization is related to a better physiological status, suggesting that the two strategies are not equivalent and that specialization consists in a ecological advantage.

Finally, our findings are also relevant for the conservation of this species and for the understanding of its ecologic role in forest ecosystems. Indeed the fact that a species with a narrow trophic niche could be able to shift on different prey categories when resources are scarce, though showing a decrease in fitness, could represent a highly adaptive trait in case of habitat fragmentation and other stressing situations that could affect the viability of *Salamandrina* populations. Lastly, the large amount of predation on Collembola suggests that *Salamandrina* could be able to regulate the abundance of these arthropods, that play a role in litter turnover in many forest ecosystem processes^29^, and that this top-down effect could have serious implications in litter retention rate and carbon capture^30^,^31^.

## Methods

### Study area and species

Fieldwork was carried out in Central Italy (41°44′50 N, 14°11′40′′ E), in two Apennine forest sites, 3 km apart, where *Salamandrina perspicillata* breeds. These sites, situated at about 900 m a.s.l., can be attributed to a Supra-Mediterranean mixed deciduous forest^32^ dominated by Beech (*Fagus sylvatica*) and Turkey Oak (*Quercus cerris*), with a limited occurrence of Hornbeam (*Carpinus betulus)*, Maple (*Acer spp.*) and Silver Fir (*Abies alba*).

This salamander is endemic to central and northern Italy^33^ and inhabits mainly shady and damp areas but also Mediterranean habitats. Adults are terrestrial and females go to water only for spawning^34^. The foraging activity of *Salamandrina* is performed only during its terrestrial phase^35^. At the study site *Salamandrina perspicillata* is the only Urodelan observed.

### Sampling of salamanders and their prey

We carried out sampling of salamanders within the early morning hours, or during light rain, when they are more active on the ground. Captured animals were processed in the field or in some cases transported to the lab for immediate treatment, in order to prevent prey digestion^36^,^37^. Total length (TL: distance from the tip of the snout to the tip of the tail) and snout-vent length (SVL: distance from the tip of the snout to the posterior margin of the cloacal slit) were measured with a caliper (precision 0.01 mm), while the body mass was measured using a digital scale (precision 0.01 g). Sex was determined by the observation of the cloaca walls^38^. Stomach flushing^39^ was performed always by the same person using a 5 ml syringe (one injection per salamander) connected to a flexible plastic tube, and stomach contents were preserved in 70% ethanol. All treated animals were returned to their capture site within few hours after capture and we did not observe mortality.

In order to investigate prey use and selection we collected samples of potential invertebrate prey at the study site in two seasons: Autumn (25–29 October 2012) and Spring (8–12 May 2013, at the end of spawning). To obtain representative data of prey availability we used three different sampling methods. Ground-dwelling invertebrates were sampled using ten pitfall traps, each one consisting of a 500 cm^3^ container, partially filled with a killing/preserving solution^40^. Pitfall traps were active for 14 days before salamanders’ sampling (between October 12 and 25 – between April 25 and May 8). Two soil cores of 1000 cm^3^ were obtained the first day of salamanders’ sampling and invertebrates were extracted using Berlese-Tullgren extractor^41^. Aerial invertebrates were sampled by ten sticky traps, active for six days before salamanders’ collection, and consisting of transparent acetate sheets (0.062 m^2^) covered with entomological glue and fixed to vegetation at 1.20 m from the soil. The positions of the traps were recorded in Autumn 2012 by flagging and, in Spring 2013, they were repositioned exactly in the same locations in order to reduce possible variations resulting from microhabitat differences.

Invertebrates obtained from environmental sampling and from stomach contents were sorted, identified, and counted using a dissecting microscope and taxonomic keys. Since invertebrates obtained with stomach flushing are partly digested, the achievable taxonomic rank is low (Order) and all invertebrates, both from stomach contents and from environmental sampling, have been determined at the Order level, annotating the life stage if ecologically relevant (e.g Lepidoptera larvae).

### Data Analysis: Is *Salamandrina* a specialist predator?

Since the first goal of our study was to confirm that *Salamandrina* is a specialized predator on Collembola, we conducted the following analysis merging together the diet data from the two populations, after appropriate analyses (see Results). Prey diversity both in the realized trophic niche and in the environment was estimated using the Shannon-Weaver (S-W) index. Possible presence of ecological sex dimorphism in diet was tested by ANOSIM^42^. The observed feeding strategy was then assessed using Costello’s^43^ modified graphical analysis^44^, which plots prey frequency of occurrence [FO; frequency of occurrence of predators feeding on prey (i)] against prey specific abundance [Pi; relative abundance of prey item (i) calculated on the total items found only in those individuals that fed on this prey category]. This method, used in dietary studies both in terrestrial and aquatic amphibians^10^,^11^,^28^, gives useful interpretation of the realized trophic niche and allows to identify the emerging trophic strategy of the population (e.g. generalist *vs* specialist).

### Data Analysis: How does *Salamandrina* select prey?

With regard to prey selection in the environment we compared the relative abundance of each prey category in the diet with the relative abundance of the same prey sampled in the environment. For this purpose we used the Vanderploeg and Scavia’s Relativized Electivity Index E*^45^, which is strongly supported^46^. This index ranges from −1 (negative selection) to 1 (positive selection), assuming a zero value for random feeding. E* was calculated only for prey categories with more than four individuals sampled in the environment. The threshold value above and below which E* was considered different from zero, was the 5th percentile of the absolute value of E*^47^. In order to determinate the effect of phenotypic variation, sex and seasonality on prey selection we used in logistic resource selection probability functions (RSPF) under a use-availability design^48^,^49^,^50^, obtaining the probability of use for each prey category, along with the effect of the covariates included in the models. The dataset for this analysis was obtained considering each prey item in the stomach of a salamander as a single predation event, and therefore we built a dataset composed by near 4000 observations of predation, along with continuous and categorical covariates, relative to individuals or season/site. Sex, seasonality, site, SVL and an index of foraging intensity (FORI – calculated as the number of ingested prey items) were used as covariates, along with the body condition index, which is basically derived from individual body mass and lenght and is considered as a good predictor of energy reserve and physiological status of the individual^51^. Among the many formulations of body condition indices, available in literature, we adopted the Scaled Mass Index (SMI) which is actually considered the best predictor of energy reserve^52^. Using the R package “ResourceSelection” we built several models for each prey category; starting from simplest models with only one covariate and adding more covariates in a stepwise approach. Models were then ranked by Akaike’s Information Criterion (AIC)^53^ taking into account that models with score differences >2 do not have the same empirical support and show substantial differences^54^, moreover the Hosmer-Lemshow goodness of fit test for logistic regression has been used to assess model fit^55^.

### Data Analysis: Is there inter-individual diet variation?

In order to assess the presence of inter-individual diet variation, and to investigate its relationship with seasonality or phenotypic variation, we analyzed the realized trophic niche using the R package “RInSp”^56^. In both seasons the trophic niche width of the population (TNW) was calculated using the equations proposed by Roughgarden^57^ and modified for discrete data^4^, using the Shannon-Weaver index as a proxy for variance. TNW has been broken down in its two components: Between Individual Component (BIC) and Within Individual Component (WIC). The ratio WIC/TNW, that ranges from 0 to 1, is a measure of inter-individual diet variation: values near 1 indicate low inter-individual diet variation, while values near 0 indicate decreasing inter-individual overlap and higher individual specialization^8^. In order to assess statistical significance of WIC/TNW we generated, through Monte Carlo resampling, 999 simulated populations from the original dataset, each one of the generated populations having a number of individual equal to the number of the real population and assigning to each individual random diet items from the population’s resource distribution, yielding to a null model corresponding to a population composed by generalists individuals^4^. The index WIC/TNW is then recalculated for each resampled dataset: the proportion of resampled populations that had index values lower than the observed one corresponds to a non-parametric p-value of the observed WIC/TNW. Another measure of individual specialization is the proportional similarity index (Psi), that describes the overlap between an individual i’s diet and the diet of the population as a whole^4^,^58^. For individuals consuming prey in direct proportion to the population as a whole, PSi will be 1 and it will decrease in case of individual specialization. In addition, we calculated the IS index which is the average of the PSi values, that represents a general measure of the individual specialization at the population level^4^,^59^. Statistical significance for this index was calculated following the same simulation approach as for the WIC/TNW index. Furthermore, in both seasons we divided the dataset in two groups: individuals that shown significant inter-individual variation (Low PSi), and individuals consuming prey in direct proportion with the population (High PSi). The threshold value of PSi below which we considered the individuals as “individual specialist” was set according to the lower confidence interval limit of the null distribution derived from 999 Monte Carlo resamplings of the original data set. We looked for differences in SVL, SMI, Shannon-Weaver diversity index, FORI and relative abundance of Collembola calculated on each stomach for these two groups, using Mann-Whitney non-parametric test.

### Ethic statement

All the experimental protocols were approved by the Italian Ministry of Environment with the authorisation number PNM-II-2012-0015691. All methods and experimental protocols were carried out in accordance with the guidelines provided by the Italian Ministry of Environment.

## Additional Information

**How to cite this article**: Costa, A. *et al.* Generalisation within specialization: inter-individual diet variation in the only specialized salamander in the world. *Sci. Rep.* **5**, 13260; doi: 10.1038/srep13260 (2015).

## Figures and Tables

**Table 1:** Prey seasonal variation in the diet of *Salamandrina perspicillata* and in the environment traps at the study site.

		**Environment**				**Diet**			
		**Autumn**		**Spring**		**Autumn**		**Spring**	
**Prey data in the environment and in the diet of** ***Salamandrina perspicillata***		**n**	**%**	**n**	**%**	**n**	**%**	**n**	**%**
*Arachnida*	Acarina	355	23.86	622	26.63	58	3.69	165	7.05
	Araneae	57	3.83	17	0.73	194	12.36	92	3.93
	Opiliones	22	1.48	5	0.21	14	0.89	6	0.26
	Pseudoscorpiones	16	1.08	5	0.21	21	1.34	20	0.85
*Miriapoda*	Symphila	1	0.07	5	0.21	0	0	0	0
	Diplopoda	16	1.08	32	1.37	5	0.32	0	0
	Chilopoda	17	1.14	12	0.51	4	0.25	1	0.04
*Hexapoda*	Protura	7	0.47	0	0	0	0	0	0
	Diplura	0	0	3	0.13	0	0	1	0.04
	Collembola	411	27.62	788	33.73	1122	71.46	1899	81.08
	Coleoptera Larvae	40	2.69	26	1.11	14	0.89	4	0.17
	Coleoptera Adults	42	2.82	81	3.47	13	0.83	24	1.02
	Diptera Larvae	6	0.40	71	3.04	27	1.72	3	0.13
	Diptera Adults	233	15.66	459	19.65	25	1.59	60	2.56
	Lepidoptera Larvae	5	0.34	22	0.94	0	0	7	0.3
	Lepidoptera Adults	28	1.88	5	0.21	1	0.06	0	0
	Hymenoptera	47	3.16	36	1.54	27	1.72	8	0.34
	Formicidae	88	5.91	18	0.77	1	0.06	4	0.17
	Embioptera	2	0.13	0	0	0	0	0	0
	Dermaptera	1	0.07	0	0	0	0	0	0
	Hemiptera	50	3.36	46	1.97	28	1.78	23	0.98
	Orthoptera	2	0.13	0	0	0	0	1	0.04
	Trichoptera	1	0.07	0	0	0	0	0	0
*Mollusca*	Gasteropoda	20	1.34	17	0.73	3	0.19	1	0.04
*Crustacea*	Isopoda	4	0.27	27	1.16	13	0.83	23	0.98
*Anellida*	Lumbricidae	14	0.94	39	1.67	0	0	0	0
*Nematomorpha*	Gordioida	3	0.20	0	0	0	0	0	0
	**Total**	**1488**	**100**	**2336**	**100**	**1570**	**100**	**2342**	**100**

**Table 2:** Comparison between groups in function of inter-individual diet variation. For definition of groups see text.

**Comparison between groups**	**Autumn**		**Spring**	
	**Group 1 Psi < 0, 73 Mean (S.D.)**	**Group 2 Psi < 0, 73 Mean (S.D.)**	**Group 1 Psi < 0, 87 Mean (S.D.)**	**Group 2 Psi < 0, 87 Mean (S.D.)**
Scaled mass index	1.33 (0.21)	1.46 (0.17)	n.s.	n.s.
Shannon-Weaver index	0.80 (0.34)	0.68 (0.25)	0.77 (0.34)	0.61 (0.05)
Evenness (J)	0.65 (0.34)	0.55 (0.17)	0.58 (0.17)	0.41 (0.11)
Foraging intensity	6.4 (4.50)	20.6 (13.2)	23.4 (11.9)	46.1 (18.9)
Relative abundance of Collembola	0.45 (0.20)	0.78 (0.08)	0.74 (0.11)	0.85 (0.05)
